# The Brain Structural-Functional Vulnerability in Drug-Naive Children With Juvenile Idiopathic Arthritis: Insights From the Hippocampus

**DOI:** 10.3389/fnhum.2022.833602

**Published:** 2022-03-18

**Authors:** Yifei Weng, Cuili Yi, Hongyan Liang, Kezhao Lin, Xiaohuang Zheng, Jihong Xiao, Haiwei Han

**Affiliations:** ^1^Department of Radiology, The First Affiliated Hospital of Xiamen University, School of Medicine, Xiamen University, Xiamen, China; ^2^Department of Pediatrics, The First Affiliated Hospital of Xiamen University, School of Medicine, Xiamen University, Xiamen, China

**Keywords:** pain matrix, multimodal MRI, juvenile idiopathic arthritis, hippocampus, SPHARM

## Abstract

**Objective:**

Leveraging an integrative multimodal MRI paradigm to elaborate on the hippocampus-derived structural and functional changes in children and adolescents with juvenile idiopathic arthritis (JIA) and to explore potential correlations within the “joint-inflammation-brain” axis during the period of central neural system (CNS) development.

**Methods:**

Twenty-one patients with JIA all completed the multimodal MRI scanning, laboratory tests, and neuropsychological assessments; meanwhile, 23 matched controls were recruited. We then harnessed the spherical harmonics with a point distribution model (SPHARM-PDM) and the ROI-to-voxel functional connectivity (FC) to measure the hippocampal shape and hippocampo-cortical FC patterns. Correlation analysis was performed to explore the potential links in neuroimaging features with disease-related indices.

**Results:**

Compared to controls, JIA patients only presented an atrophic tendency in the posterior part of the bilateral hippocampus. The hippocampo-cortical FC revealed the between-group divergences mainly located at the pain matrix, striatum, and temporal lobe. Remarkably, the enhanced FC between the right hippocampus and postcentral cortex is positively correlated with the disability index, while the weakened FC of right anterior hippocampus with right insula and that of left posterior hippocampus with left superior temporal gyrus was inversely related to the erythrocyte sedimentation rate and anxiety status, separately.

**Conclusion:**

As with macroscopic damages, the altered functional-connectome patterns of the hippocampus in JIA patients might be more sensitive to detect the early neuropathological changes. Moreover, the functional disturbances were demonstrated associated with the physical disability, inflammation, and emotional status. These findings may enlighten us on the underlying neuropathological mechanism of CNS comorbidities in JIA.

## Introduction

Juvenile idiopathic arthritis (JIA) is a prevalent and highly heterogeneous pediatric rheumatic disease mainly affecting children and adolescent younger than 16 years old (Prakken et al., 2011). It was characterized as several symptoms, including joint swelling, stiffness, recurrent pain; however, it remains unclear etiology and has no radical cure. The chronic inflammation condition in patients with JIA would damage the inflammatory-molecular messengers and affect neural activities of the peripheral and central nervous system (CNS), which is evidenced by a plethora of neurological and psychiatric sequelae, such as pain central sensitization, depression, anxiety, and sleeping disorders (Hanns et al., 2018; Arnstad et al., 2020). On top of that, the long-term revolving around pain, disability, fatigue, and unpredictable physical discomforts would also severely affect the academic performance and social activity involvement of the JIA children and adolescents (Granjon et al., 2021), therefore, leading to a high risk of aggravating mental problems. Accordingly, besides the medical attention for physical manifestations, additional psychological intervention should also be considered; meanwhile, a better understanding of the mechanism of neural dysfunction in JIA would be very instructive for guiding therapeutic strategies.

Recently, the concept of “joint-brain axis” was brought forward to express the high predisposition to develop neuropsychiatric comorbidities and cognitive decline across rheumatic-spectrum diseases, especially widely investigated in animal models (Andersson et al., 2018) and adults with rheumatoid arthritis (RA) (Felger and Lotrich, 2013; Schrepf et al., 2018; Suss et al., 2020). Systemically increased levels of inflammatory cytokines, such as tumor necrosis factors-α (TNF-α), interleukin-1β (IL-1β), interleukin-6 (IL-6), and cytokines products in the CNS microenvironment emerge as risk factors for neurological symptoms. The hippocampus, a complex component of the brain, might be one of the first regions in response to inflammatory damage due to the enrichment of inflammatory-molecular factors and receptors (Felger and Lotrich, 2013; Andersson et al., 2018). As an essential component of the limbic system, the hippocampus is also dominantly in charge of memory and emotion, whereas its aberrance would have negative consequences, such as depression, anxiety, fatigue, and declined cognitive functions. Based on emerging animal studies on JIA, chronic inflammation would increase glutamatergic neurotransmission but down-regulate the expression of Brain-Derived Neurotrophic Factor (BDNF) in the hippocampus and then alter the dendritic remodeling and neurogenesis, especially involving the dentate gyrus (McEwen et al., 2015). Consequently, medication treatment for decreasing inflammatory levels might help improve neuroplasticity and neuropsychiatric symptoms in patients with JIA (Korte-Bouws et al., 2019; Poutoglidou et al., 2021). To provide enlightenment on translating between experimental research and clinical practice, the human hippocampal research using noninvasive technology is thus essential for exploring the neural disturbances at the early stage of JIA.

With neuroimaging techniques springing up, integrated structural and functional magnetic resonance imaging (fMRI) could help us non-invasively reveal the neural disturbances in the conditions of JIA, for instance, the decreased cortical thickness of insula and altered global functional connectivity (FC) (Upadhyay et al., 2021), while there was little study focused on the hippocampus. As a key cognition-related component in human brain, solid standpoints have been proposed to describe how the function is distributed along the anterior-posterior axis of the hippocampus and pictured the corresponding functional organization and specialization of neural activities in humans (Przezdzik et al., 2019). Neuroscientists demonstrated that the cortical afferents to the hippocampus were correlated with different segmentations along the long axis. A functional gradient analysis reported that the anterior part might have greater connectivity to default mode, limbic and somatomotor areas, while the posterior part exhibits more connectivity, especially with visual and dorsal/ventral attention networks (Vos de Wael et al., 2018). Accumulating fMRI literature has further manifested that the hippocampus would show excessive activities in subjects with high sensitivity to pain (Ziv et al., 2010), which characteristic is also demonstrated in JIA patients (Schanberg et al., 1997; Moriarty et al., 2011). However, little is known about how hippocampal subdivisions and neural circuits interact to produce emotion and cognition responding to painful stimuli, let alone research focused on JIA. Wherefore, a refined delineation of structural and functional properties targeting hippocampal subdivisions is imperative for better understanding the neuropathological mechanism underlying cognitive dysfunction of JIA.

Hence, we leveraged patient-specific multiscale MRI approach integrating morphology and functional connectivity to investigate the hippocampal anomalies in JIA patients, which might help extend the knowledge of neural substrates of cognitive changes. Additionally, the cognitive status of JIA could not be encapsulated by any single indicator, so that comprehensive estimations, including physician assessment, laboratory test, and neuropsychological evaluation, are also necessary. Our study aims to put a significant step forward in understanding the joint-brain axis implicated by the human hippocampus, which should play an essential role in the guidance of clinical therapy and mental care for JIA patients.

## Materials and Methods

### Participants

A total of 25 JIA children and adolescents were consecutively recruited from the Pediatric Clinic of The First Affiliated Hospital of Xiamen University, China, PR. All patients were drug-naïve and firstly diagnosed with JIA according to the International League of Associations for Rheumatology (ILAR) classification, and also informed by self-report complaints, medical imaging evidence, and laboratory blood tests [i.e., erythrocyte sedimentation rate (ESR) and C-reactive protein (CRP)]. Patients were also compared with 23 age- and sex-matched healthy controls (HCs). Before MRI scanning, participants were assessed by a series of validated neuropsychological and clinical scales: (1) Juvenile Arthritis Disease Activity Score (JADAS-27) (Tibaldi et al., 2020) and Childhood Health Assessment Questionnaire (C-HAQ) (Landgraf et al., 1996; Ruperto et al., 2001) was chosen to assess the diseased severity and the life impact of the disability; (2) The Depression Self-Rating Scale (DSRS) (Birleson, 1978) and The Screen for Child Anxiety Related Emotional Disorders (SCARED) (Birmaher et al., 1997) were selected to evaluate the emotional status of both children and adolescent with JIA and HCs, and those who were younger than eight (6–8 years old in this study) were reported by their parents. (3) For JIA patients, the Visual Analog Scale (VAS) (Johnson, 2001) was also selected to measure the intensity of the pain involving joint discomfort.

Further exclusive criteria were as follows: (1) either younger than six or older than sixteen years old; (2) left-handedness; (3) with mass lesion; (4) with history of brain surgery; (5) with significant physical or psychiatric conditions, (6) with excessive head translation or rotation parameters (>2.5 mm or 2.5°), or (7) with MRI contraindications. This study was carried out according to the declaration of Helsinki and approved by the ethics committee of The First Affiliated Hospital of Xiamen University (No. XMYY-2021KYSB011). All participants gave written informed consents before the neuropsychological tests and MRI scanning.

### Magnetic Resonance Imaging Acquisition and Data Preprocessing

Resting-state functional MRI scans were acquired on a 3.0-Tesla MR scanner (TIM Trio, Siemens Medical Solution, Erlangen, Germany). All participants were instructed to lay supine, stay still, awake, and keep their eyes closed during MRI scanning. Using 2D echo-planar blood oxygen level-dependent (BOLD) imaging, we acquired resting-state functional MRI (rs-fMRI) data with following parameters: 30 slices with no intersection gap; repetition time (TR) = 2000 ms; echo time (TE) = 30 ms; flip angle, 90°; field of view (FOV) = 240 mm × 240 mm; 200 volumes; slice thickness = 4 mm; 3.75 mm × 3.75 mm × 4 mm voxel size; the total acquisition time = 346s. High-resolution 3D anatomical T1-weighted images (T1w-3D-MPRAGE) were also collected using following parameters: 191 slices; TR = 2300 ms; TE = 2.98 ms; flip angle = 9°; FOV = 256 mm × 256 mm; 1 mm × 1 mm × 1 mm voxel size. Two experienced radiologists (HH and WY) reviewed the routine T1w and T2-FLAIR images and then excluded the patients with any structural anomaly (i.e., congenital brain malformations, tumor, ischemia or hemorrhage, and hippocampal sclerosis).

Subsequently, imaging preprocessing was conducted using a series of established software, FSL (v5.0.9^1^) (Patenaude et al., 2011) and DPABI^2^ (Yan et al., 2016). The detailed preprocessing procedures were as follows: (1) T1WI images were, in brief, input and undergone image reorientation, skull stripping, cortical and subcortical segmentation, and standard space co-registration; (2) For rs-fMRI data, the procedures of the first ten time-points removals, head-motion estimation and correction for Friston-24 parameters, slice timing correction, spatial normalization, and alignment to standard MNI space into 3 mm x 3 mm x 3 mm resolution voxels were orderly executed. The preprocessed functional data were then conducted with band-pass filtering (0.01–0.1 Hz) and spatial smoothing with a 6 mm full width at half-maximum (FWHM) Gaussian kernel. We also statistically corrected for average white matter, cerebrospinal fluid, and mean global signals.

### The Construction of Hippocampus Surface Using Spherical Harmonics With a Point Distribution Model Method

The individually segmented hippocampus images on MNI152 space were converted to surface meshed and parameterized according to spherical harmonics with a point distribution model (SPHARM-PDM) (Chung et al., 2010). We generated the left and right hippocampal template with ∼4k vertices across all HCs, where the individual surface was aligned based on their intrinsic shape features. We then calculated the displacement vectors between the individual’s surface and the template along the average direction of each face of triangle mesh, indicating inward or outward deformation of each patient relative to HCs.

### The Construction of Region of Interests-to-Voxel Functional Connectivity Matrix

According to the existed hippocampus atlas, the bilateral ROIs of the hippocampus, which regions we were highly concerned about in the current study, were obtained from Automated Anatomical Labeling (WFU Pick Atlas Tool version 3.0^3^) (Maldjian et al., 2003). These seed regions were created in the standard MNI space and were subsequently separated into three sections (including anterior, middle, and posterior part) along the A-to-P axis as in previous studies (Chen and Etkin, 2013; Zheng et al., 2021). For supplementing the structural analysis, we individually measured the number of voxels in each ROI. We then applied the hippocampal seed regions to extract the time series, and the ROI-to-voxel hippocampo-cortical FC was calculated with Pearson correlation coefficient. The individual FC matrix then underwent Fisher r-to-z transformation prior to further statistical analysis.

### Statistical Analysis

SPSS 22.0 (SPSS INC, Chicago, IL, United States) was used to analyze the demographic, clinical, and neuropsychological data. The Chi-square test was applied to evaluate gender difference between groups. The homogeneity of variance was examined by the Bartlett test. Then the normally distributed (i.e., age and DSRS) and non-normally distributed (i.e., SCARED) materials were, respectively, assessed by the two-sample *t* test and the Mann-Whitney *U* test. The disease-related scores, such as JADAS, C-HAQ, VAS, and laboratory indices, were also displayed with mean and range.

Surface-based hippocampal shape analysis was carried out using both Hotelling’s T-square statistic and SurfStat^4^ (Chung et al., 2010) based on MATLAB (R2017b, The Mathworks, Natick, MA, United States). The general linear model for SurfStat was set as follows: Model1 = β0 + β1×Sex + β2×Age + β3×*Brainvolume*. For the FC analysis, the voxel-wise hippocampus statistical analysis was carried out by the tool established in DPABI while also controlling for age, sex, and brain volume. All the results were performed the multiple comparison correction using a permutation test (5000 permutations) with threshold-free cluster enhancement strategy (TFCE) as implemented in the DPABI statistical toolbox. The threshold for the final display was set at the level of two-tailed *P* < 0.05 and the cluster size of 90 (Chen et al., 2018).

To assess the potential association of altered neuroimaging features with clinical and cognitive signs, characteristics of regions with group differences were separately extracted and correlated with corresponding clinical estimation, neuropsychological performance, and laboratory indicators, with a significant threshold level of *P* < 0.05. It should be noted that the mean FC values were separately extracted from spherical ROIs (*r* = 6 mm) (Fox et al., 2005), centering at the spots with peak *t* value from those regions showing statistical significance. All results were visualized with BrainNet Viewer^5^ (Xia et al., 2013).

## Results

### Demographic, Neuropsychological, and Clinical Data

The demographics, clinical estimates, neuropsychological and laboratory data of JIA patients and HC are shown in Table 1. Four patients were excluded because of excessive head motions. There was no participant showing structural abnormality on the routine MRI examination. The final sample of patients with JIA included following subtypes: 29% (*n* = 6) oligoarticular, 24% (*n* = 5) polyarticular, 38% (*n* = 8) enthesitis-related, and 9.0 % (*n* = 2) systemic arthritis. No group differences in gender, age was found between the two groups (*P* > 0.05). JIA patients showed relatively higher score of depression (*P* < 0.05) to HCs. As for disease-related blood indicators, we found the increased group-level of ESR and CPR in JIA.

**TABLE 1:** Demographics, neuropsychological estimates and laboratory data.

	JIA	HC	*P*
	(*n* = 21)	(*n* = 23)	
Sex (M/F)	9/12	7/16	0.732^a^
Age (years)	8.95 ± 2.36	9.22 ± 2.32	0.709^b^
DSRS	9.24 ± 5.12	6.26 ± 2.70	0.019^b^*
SCARED	16.67 ± 14.62	10.30 ± 6.09	0.258^c^
Duration (months)	9.69 ± 22.69	–	–
**Disease-related index (mean, range)**		–	–
JADAS	15.71 (5.0 – 39.20)	–	–
C-HAQ	0.54 (0.0 – 2.25)	–	–
VAS	30.48 (0.0 – 80.0)	–	–
**Laboratory tests**			
ESR (mm/h)	16.22 (3.0 – 78.0)	–	–
CRP (mg/L)	9.99 (<0.5 – 103.17)		

*^a^Chi-square test.*

*^b^Two sample t-test.*

*^c^Mann-Whitney U test.*

*P < 0.05.

*JIA, juvenile idiopathic arthritis; HC, healthy control; M, male; F, Female; DSRS, The Depression Self-Rating Scale; SCARED, The Screen for Child Anxiety Related Emotional Disorders; JADAS, Juvenile Arthritis Disease Activity Score; C-HAQ, Childhood Health Assessment Questionnaire; VAS, Visual Analog Scale; ESR, erythrocyte sedimentation rate; CPR, C-reactive protein (CRP). The demographic and neuropsychological data (i.e., age, DSRS, SCARED, and duration) were displayed with mean ± standard deviation, and the disease-related indices (i.e., JADAS, C-HAQ, VAS, and laboratory tests) were displayed with mean and range.*

### The *in vivo* Overview of Hippocampal Shape

**FIGURE 1:**
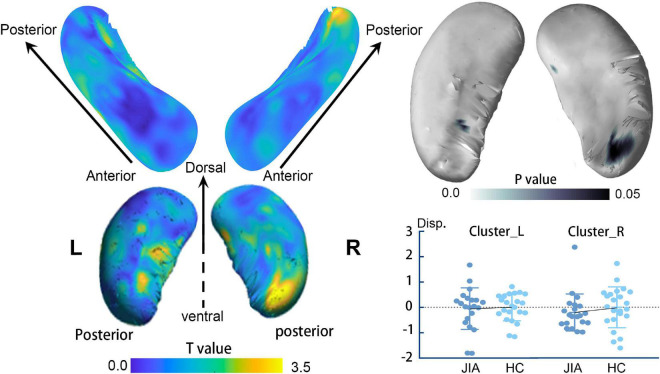
The surface-based hippocampus shape analysis. The left panel shows the t value of the group comparison result at each vertex on the surface template. The right panel displays the projected *p*-value on the template. The clusters in the dark blue indicate the shrunken regions with *p* < 0.05; however, these findings could not survive from multiple comparisons at a family-wise level of 0.05. The plot chart represents the individual mean value of the displacements in each cluster. The error bar represents the standard error of the mean. L, left; R, right; JIA, juvenile idiopathic arthritis; HC, healthy controls.

**FIGURE 2:**
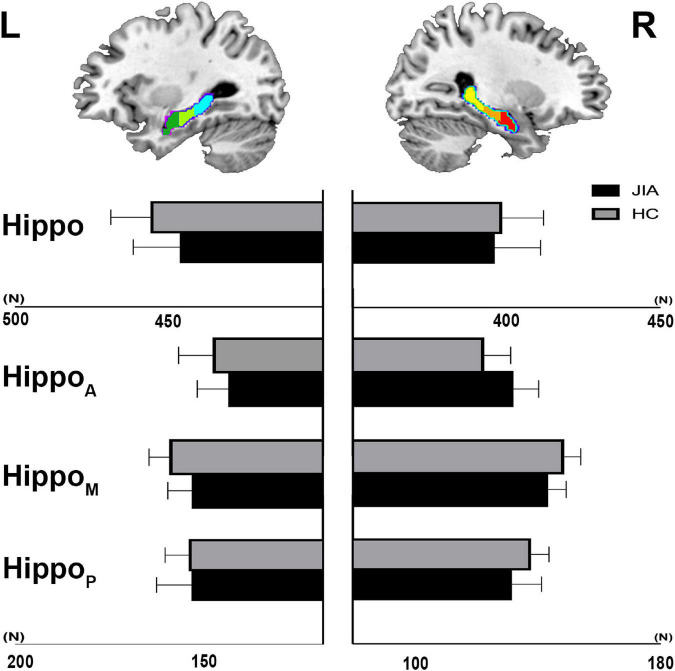
The volumetry of hippocampus and its subdivisions. The group level of mean voxel number within the hippocampus mask and its subdivisions is shown in the bar chart. The error bar represents standard deviation. L, left; R, right; JIA, juvenile idiopathic arthritis; HC, healthy controls; Hippo, hippocampus; HippoA, anterior subdivision of hippocampus; HippoM, middle subdivision of hippocampus; HippoP, posterior subdivision of hippocampus.

The direct comparisons between groups revealed the shrunken trend in the posterior part of the bilateral hippocampus (Hostelling’s T-square test, *P* < 0.05; left cluster size = 56 vertices, right cluster size = 152 vertices). The statistical power of the results was relatively low that could not survive from the family-wise error (FWE) correction. Although, there was a quite marked cluster on the right posterior hippocampus (Figure 1). To supplement the surface result, the volumetry analysis on different subdivisions of the hippocampus revealed that both left and right hippocampus showed smaller mean volume sizes in JIA group relative to HCs, except for the right anterior hippocampus (Figure 2). However, we did not detect any statistical significance in the volumetry analysis after the multiple comparison correction.

### The Group Difference in Subdivided Hippocampo-Cortical rs-Functional Connectivity Analysis

**FIGURE 3:**
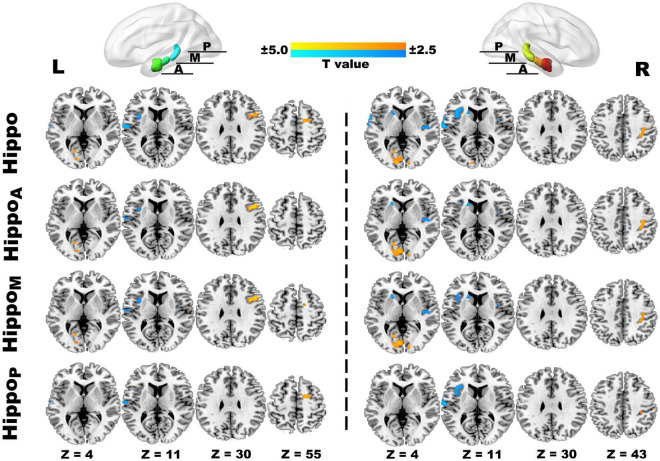
The ROI-to-voxel rs-FC differences in the hippocampus and its subdivided parts between JIA and HCs. The first panel separately displays five clusters (L) and six clusters (R) showing rs-FC differences between children with JIA and HCs. The cool color indicates regions with decreased rs-FC, and those with warm color shows increased rs-FC in JIA compared to HCs. The color bar indicates the T score. Further details are presented in Table 2. The second to fourth panels show the dominant distributions of the altered rs-FC involving different hippocampus subdivisions. All group comparisons are performed with the two-sample t test controlling for age and sex (*p* < 0.05, 5000 permutations, TFCE corrected). L, left; R, right; A, anterior; M, middle, P, posterior; Hippo, hippocampus; HippoA, anterior subdivision of hippocampus; HippoM, middle subdivision of hippocampus; HippoP, posterior subdivision of hippocampus.

Comparing to HCs, our study primarily detected the anomalies of rs-FC hippocampus patterns in JIA, which mainly involved in pain matrix (Morton et al., 2016) (i.e., insula, primary and secondary somatosensory cortices, and frontal cortices), striatum, and temporal lobe (Figure 3 and Table 2). In detail, the right hippocampus showed reduced FC with the bilateral insula, left caudate, and left superior temporal gyrus, as well as increased FC with ipsilateral postcentral gyrus (PostCG) and bilateral occipital lobe; while the left hippocampus shared quite similar altered FC patterns such as decreased FC with the ipsilateral insula, superior temporal gyrus and increased FC with the bilateral occipital lobe. Additionally, the left hippocampus exhibited increased FC with the frontal lobe, including the opercular part of the inferior frontal gyrus (IFGoper) and superior frontal gyrus (SFG). The results of hippocampus subdivisions were further masked by the FC results of the ipsilateral hippocampus to specify the dominant contributions from each subdivision. We found the decreased hippocampal FC with ipsilateral insula and contralateral striatum were contributed by the anterior and middle subdivisions. In addition, the enhanced connections of the left hippocampus with ipsilateral IFGoper and SFG were also contributed by different subdivisions. However, the decreased FC between left hippocampus and ipsilateral STG, increased FC between right hippocampus and ipsilateral PostCG, as well as the extended FC decreases involving right hippocampus with contralateral insula could be observed in every subdivided FC map.

**TABLE 2:** The dominant brain regions show altered rf-FC with the hippocampus and its subdivisions.

Brain region (AAL)	MNI (x, y, z) Coordinate	Cluster size (mm^3^)	JIA	HC	Peak *t* intensity
				
			Mean FC values		
**Hippo (R)**					
Insula (R)	39, −15, 2	172	0.11 ± 0.15	0.22 ± 0.12	–3.13
Insula (L)	−39, 15, 9	79	0.03 ± 0.12	0.15 ± 0.13	–3.62
PostCG (R)	33, −33, 39	41	0.02 ± 0.13	−0.12 ± 0.13	3.97
Caudate (L)	−17 17 8	52	0.08 ± 0.15	0.23 ± 0.18	–3.01
STG (L)	−66, −12, 15	60	−0.07 ± 0.12	0.09 ± 0.16	–4.49
Occipital (L/R)	3, −72, −6	394	0.13 ± 0.09	−0.04 ± 0.14	3.99
**HippoA (R)**					
Insula (R)	41, −2, 9	103	0.10 ± 0.20	0.18 ± 0.17	–3.34
Insula (L)	−39, 15, 9	34	0.08 ± 0.10	0.16 ± 0.12	–2.10
PostCG (R)	37, −22, 41	39	0.13 ± 0.13	−0.03 ± 0.13	4.01
Caudate (L)	−17 19 9	49	0.03 ± 0.15	0.18 ± 0.15	–3.75
Occipital (L/R)	3, −72, −6	320	0.08 ± 0.13	−0.04 ± 0.14	3.27
**HippoM (R)**					
Insula (R)	39, −3, 12	108	0.08 ± 0.22	0.16 ± 0.17	–2.43
Insula (L)	−38, 15, 8	33	0.08 ± 0.12	0.17 ± 0.13	–3.05
PostCG (R)	33, −33, 39	34	0.12 ± 0.12	−0.01 ± 0.15	3.49
Caudate (L)	−20, 17, 9	32	0.03 ± 0.15	0.18 ± 0.16	–3.43
Occipital (L/R)	−3, −78, 3	253	0.12 ± 0.14	−0.02 ± 0.16	3.83
**HippoP (R)**					
Insula (L)	−39, 7, 9	37	−0.02 ± 0.11	0.10 ± 0.16	–2.70
PostCG (R)	33, −33, 39	38	−0.01 ± 0.16	−0.12 ± 0.13	2.72
STG (L)	−63, −13, 10	37	−0.13 ± 0.15	0.02 ± 0.12	–3.40
**Hippo (L)**					
Insula (L)	−37,8, 11	154	0.01 ± 0.13	0.16 ± 0.18	–3.23
IFGope (R)	45, 15, 24	53	0.03 ± 0.12	−0.10 ± 0.11	3.56
STG (L)	−60, −13, 11	130	−0.02 ± 0.13	0.16 ± 0.18	–3.79
SFG (R)	24, −3, 60	96	−0.01 ± 0.16	−0.14 ± 0.11	3.55
Occipital (L/R)	−18, −66, 0	227	0.09 ± 0.10	−0.02 ± 0.11	4.06
**HippoA (L)**					
Insula (L)	−37, 5, 11	33	0.08 ± 0.12	0.20 ± 0.12	–2.48
IFGoper (R)	45, 15, 30	50	0.05 ± 0.11	−0.08 ± 0.09	4.46
STG (L)	−58, −15, 11	21	0.06 ± 0.14	0.16 ± 0.16	–2.62
Occipital (L/R)	−18, −66, 0	190	0.12 ± 0.12	−0.02 ± 0.12	4.21
**HippoM (L)**					
Insula (L)	−37, 9, 8	58	0.07 ± 0.13	0.20 ± 0.19	–2.83
IFGoper (R)	45, 15, 27	52	0.08 ± 0.11	−0.05 ± 0.10	4.56
STG (L)	−58, −11, 13	36	0.05 ± 0.14	0.18 ± 0.18	–2.70
Occipital (L/R)	−15, −66, 0	138	0.14 ± 0.14	−0.01 ± 0.11	4.20
**HippoP (L)**					
STG (L)	−60, −13, 11	105	−0.10 ± 0.14	0.03 ± 0.14	–3.12
SFG (R)	27, −9, 60	50	0.01 ± 0.09	−0.10 ± 0.15	3.43

*ROI, region of interests; MNI, Montreal Neurological Institute; JIA, juvenile idiopathic arthritis; HC, healthy controls; AAL, automated anatomical labeling; FC, functional connectivity; Hippo, hippocampus; HippoA, anterior subdivision of hippocampus; HippoM, middle subdivision of hippocampus; HippoP, posterior subdivision of hippocampus; ITG, Inferior temporal gyrus; PostCG, postcentral gyrus; STG, superior temporal gyrus; IFGoper, opercular part of inferior frontal gyrus; SFG, superior frontal gyrus; R, right; L, left.*

### The Correlations Among Hippocampo-Cortical Functional Connectivity, Clinical Measurements, Laboratory Tests, and Neuropsychological Assessments

**FIGURE 4:**
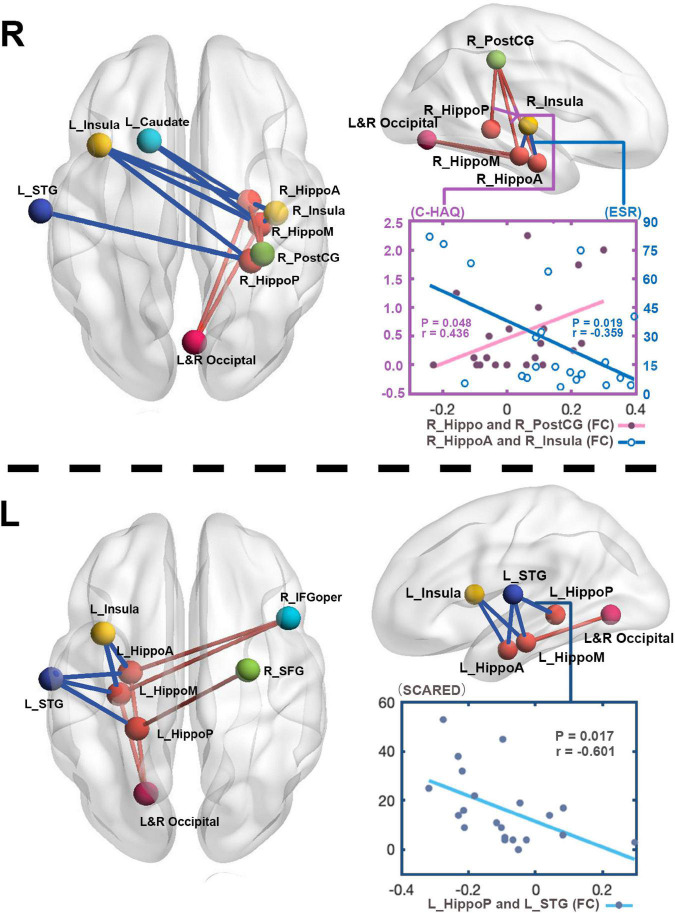
The relations between altered FC and clinical measurements. To directly observe the changed FC patterns, we aggregated all the FC changes of each hippocampus into one connectivity map. The FC between right hippocampus and right PostCG in patients positively correlates with C-HAQ scores (*r* = 0.436, *P* < 0.05), and that between right anterior hippocampus and right insula negatively correlates with ESR (*r* = –0.359, *P* < 0.05). The FC values between left posterior hippocampus and left STG in patients negatively correlate with SCARED scores (*r* = –0.601, *P* < 0.05). R, right; L, left; JIA, juvenile idiopathic arthritis; HC, healthy controls; C-HAQ, Childhood Health Assessment Questionnaire; ESR, Erythrocyte Sedimentation Rate; SCARED, The Screen for Child Anxiety Related Emotional Disorders; Hippo, Hippocampus; HippoA, anterior subdivision of hippocampus; HippoM, middle subdivision of hippocampus; HippoP, posterior subdivision of hippocampus; ITG, inferior temporal gyrus; PostCG, postcentral gyrus; STG, superior temporal gyrus; IFGoper, opercular part of inferior frontal gyrus; SFG, superior frontal gyrus.

**FIGURE 5:**
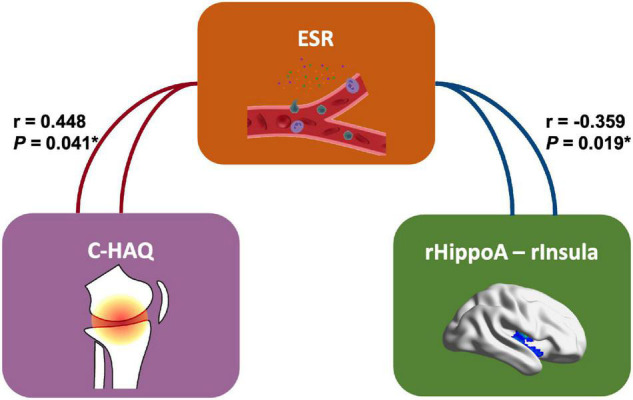
The correlation schema on joint-inflammation-brain axis in JIA. This panel presents the potential relationships inside the joint-inflammation-brain axis involving the hippocampal-based FC (right anterior hippocampus – right Insula). C-HAQ, Childhood Health Assessment Questionnaire; ESR, erythrocyte sedimentation rate; rHippoA, right anterior hippocampus, rInsula, right Insula.

Our finding further revealed the associations of changed functional activities with the physical and mental manifestations reported by patients with JIA (Figure 4). In JIA group, the FC between right hippocampus and right PostCG was positively correlated with C-HAQ scores (*r* = 0.436, *P* = 0.048), and that between right anterior hippocampus and right insula was negatively correlated with ESR (*r* = −0.359, *P* = 0.019); meanwhile, the ESR also found positive correlation with the C-HAQ (*r* = 0.448, *P* = 0.041). We also observed that the FC between the left posterior hippocampus and left STG was negatively correlated with SCARED scores (*r* = −0.601, *P* = 0.017). We then summarized the abovementioned correlations into a potential link inside the joint-inflammation-brain axis (Figure 5), although we failed to detect the mediated effect among the disease manifestations, inflammatory indices, and hippocampal-based neural activities (MacKinnon et al., 2000).

## Discussion

Our findings indicated the functional susceptibility of the hippocampo-cortical network in JIA patients, which were mainly involved in the pain matrix, striatum, and adjacent temporal lobe. Except for the functional changes, we also detected a tendency of structural contraction in the bilateral posterior hippocampus. Findings of more marked functional perturbances supported the notion that nociceptive CNS changes in the context of JIA is associated with more severe whole-brain anomalies rather than a focal damage. Notably, the enhancement of FC between the hippocampus and ipsilateral primary and secondary somatosensory cortices is correlated with the severity of daily dysfunction; and furthermore, the changes of the posterior hippocampal-based functional-connectome anomaly was related to the anxiety level. All these findings implied that the hippocampus, especially its altered FC patterns with pain-related brain regions, might be the sensitive neuroimaging biomarkers for detecting the early brain disturbances affected by the disease and inflammatory status.

### Spherical Harmonics With a Point Distribution Model Analysis Offers an *in vivo* Overview of Macroscale Hippocampal Shape in Juvenile Idiopathic Arthritis

Deriving a 3D high-resolution surface modeling method, we statistically mapped the tendency of bilateral hippocampal atrophy in JIA patients. Compared with old fashion voxel-based morphometry (Bookstein, 2001), SPHARM-PDM allows statistical inference at the resolution of individual space, benefiting from the increase of sensitivity and anatomic precision (Styner et al., 1071). We finally detected the subtly shrunken trend in the bilateral posterior hippocampus, although our structural findings did not show the rigidly statistical significance. This finding was consistent with previous rodent studies that mimicked local and systemic inflammation conditions, which ultimately found the structural alteration and impaired hippocampal neurogenesis (Ekdahl et al., 2003; Monje et al., 2003). In brief, chronic peripheral inflammation would trigger the alteration of neurotransmitter transduction in the hippocampus, mainly involving glutamatergic and serotonergic signaling, wherefore dysregulating the homeostatic function of astrocytes and then leading to the neuropsychiatric symptom (Suss et al., 2020). Our observation further extended the prior finding that chronic peripheral inflammation would induce complex central responses and probably would cause structural changes in the human brain.

### rs-FC Analysis Reveals the Rearrangements in Subdivided Hippocampo-Cortical Connectome of Juvenile Idiopathic Arthritis

A previous study on JIA children has observed CNS circuitry anomalies with integrating structural and functional MRI during both resting- and evoked- status (Upadhyay et al., 2021). To supplement the prior findings, we used the seed-based FC analysis, laying heavy emphasis on the prior hypothesis that the hippocampus would be a sensitive probing to identify the neural changes in JIA, which may better focus on the cognitive-related neural hubs and networks reorganization.

In the present study, increased hippocampal FC was found in postCG and lateral prefrontal cortices in JIA patients. As we know, chronic inflammatory pain is well-recognized as a significant symptom in JIA patients and associated with multiple components activation in CNS (i.e., sensory, emotion, cognition, and behavioral elements) (Yang and Chang, 2019). A numbers of pain quantitative studies based on self-reported pain, thermal, or pressure pain thresholds have demonstrated that there were a great proportion of JIA children would suffer from pain hypersensitivity (Leegaard et al., 2013; Arnstad et al., 2020). The increased connectivity between the hippocampus and primary sensorimotor cortices (postCG) may reflect the arousal of the sensorimotor processing and the hypersensitivity to the noxious stimulus (Price, 2000). We furthermore observed the FC strength between the entire hippocampal region and postCG was positively correlated with the disability index of C-HAQ. The underlying correlation indicated that the perception of physical dysfunction could bring about neural disturbances at the early stage of pain perception, regardless of hippocampal subdivisions. We also detected the increased FC between different hippocampal subfields and varied lateral prefrontal cortices. In detail, the left anterior and middle part of the hippocampus has an enhanced connection with IFG (Brodmann area 44/45/48), whereas the posterior hippocampus has increased FC with SFG (Brodmann area 6). The lateral prefrontal cortex is closely connected with temporal and parietal regions responsible for the implementation of multiple cognitive behaviors as working memory, attentional selection, and planning (Joni, 2019). The discrimination of FC patterns between lateral prefrontal cortices and hippocampal subfields may rely on the anatomically adjacent structures, neural fiber bindings, and the complex activities within neural circuits in response to pain and inflammation (Ong et al., 2019). For instance, in the process of pain modulation, the lateral part of the frontal cortex (such as areas 6) would participate in the projections of cortices to the periaqueductal gray (PAG), where is the primary control center for descending pain regulation and pain relief (Periaquiductal, 2011); while the IFG that borders on the insula have suggested involved in pain-related empathic processing and interoceptive (the ability to sense the physiological condition of the body) (Li et al., 2021). Consequently, these abnormal activations might lower the pain tolerance, boost frustrating expectations of joint pain, and then strengthen the harmful experience shaping in children and adolescents with JIA.

We additionally identified the decreased hippocampal FC with insula in the JIA cohort and its association with the inflammatory level (ESR) — a laboratory index also showed association with JIA physical disability. In support of our findings, Upadhyay et al. (2021) have recently revealed the reduced cortical thickness of insula in JIA patients, and the decreased FC between anterior insula and medial prefrontal cortex was correspondence with greater clinical pain intensity. The insula is a significant center engaged in the interception or self-referential thinking for pain processing, wherefore the decoupling of the insula with hippocampus or other cognitive networks might facilitate the subjective sense of pain in JIA patients (Gu et al., 2013; Uddin et al., 2017). Moreover, the hippocampus also exhibited decreased FC with the caudate and temporal lobe. It has been demonstrated that the hippocampus possesses abundant neural fibers with subcortical nuclei and temporal lobe, and also a participant in the regulation of limbic hypothalamic-pituitary-adrenal axis, whose functions could be modulated by endogenous opioid peptides and subserve the physiological adaption to stress (Rubinstein et al., 1996). Meanwhile, the pain processing regions, including temporal and caudate, have been reported accompanied by changes in the endogenous opioid system during the painful period (Zubieta et al., 2001; Sprenger et al., 2005). Accordingly, the weakened FC of the hippocampus with striatum and temporal lobe might result from the disturbed neurotransmitter homeostasis, which plays a pivotal role in the altered neuroplasticity and development of mental disorders (McEwen et al., 2015). The assumption could be further evidenced by our finding that the FC of the left posterior hippocampus and left STG was related to the level of anxiety.

### Highlights and Limitations

The current study has some highlights and unfulfillments. To our best knowledge, neuroimaging research on JIA children and adolescents is limited. This is the first neuroimaging study focused on the drug-naïve JIA cohort. In our sample, all the participants were absent of medication treatment before the neuropsychiatric assessments and MRI scanning. Medications such as corticosteroids for systemic JIA were already demonstrated to disrupt the brain structures, including the hippocampus and frontal lobe (Ciriaco et al., 2013), wherefore our studies could diminish such confounding factors. Secondly, the study on the certain relationships between arthritis and cognitive behavior has not been investigated in-depth, especially targeting young and developing CNS. We conducted novel fMRI methods to specifically elucidate the neural anomalies of the hippocampus and its subdivisions in JIA patients, which could help broaden our understanding of the “joint-brain axis” in the arthritis-related spectrum. As for limitations, the sample size was relatively limited, and the enlargement of the JIA group is necessary for checking the repeatability. Besides, our research included diverse subtypes, so the variances between different JIA groups remain explored. More importantly, in this preliminary study, although the effect of brain volume has been considered into morphological analysis, a follow-up study is needed to track the actual developing trajectory in these JIA children.

## Conclusion

Collectively, the altered FC in hippocampo-cortical networks support the pathophysiological assumption that hippocampal-derived neural activities are altered in patients with JIA. The inflammatory status is apparently not only related to the physical discomfort but affects cognitive behavior and mental health. Furthermore, we found the hippocampal functional activities were more susceptible than the morphology in the JIA group, which might be a sensitive probing for detecting the neural disturbance and guiding early clinical and mental intervention. Although the exact relationship between the immune status, peripheral inflammation, and neuroinflammation remains unclear, our study might shed some light on the pathogenesis of CNS comorbidities in the context of JIA.

## Data Availability Statement

The original contributions presented in the study are included in the article/supplementary material, further inquiries can be directed to the corresponding authors.

## Ethics Statement

The studies involving human participants were reviewed and approved by the Ethics Committee of the First Affiliated Hospital of Xiamen University (No. XMYY-2021KYSB011). Written informed consent to participate in this study was provided by the participants’ legal guardian/next of kin.

## Author Contributions

YW and CY were involved in the literature review, neuroimaging data analysis, and manuscript writing. HL edited the manuscript. KL and XZ performed clinical data analysis and aided in writing the manuscript. JX was in charge of the clinical and laboratory data analysis, literature review, and modification of the manuscript. HH designed the study and assisted in writing the manuscript. All authors were qualified for authorship according to the ICMJE criteria, and read and approved the final manuscript.

## Conflict of Interest

The authors declare that the research was conducted in the absence of any commercial or financial relationships that could be construed as a potential conflict of interest.

## Publisher’s Note

All claims expressed in this article are solely those of the authors and do not necessarily represent those of their affiliated organizations, or those of the publisher, the editors and the reviewers. Any product that may be evaluated in this article, or claim that may be made by its manufacturer, is not guaranteed or endorsed by the publisher.

## Footnotes

1
https://fsl.fmrib.ox.ac.uk/fsl

2
http://fmriprep.org

3
https://www.nitrc.org/projects/wfu_pickatlas/

4
http://www.math.mcgill.ca/keith/surfstat

5
http://www.nitrc.org/projects/bnv/
